# User-Centered Design of Learn to Quit, a Smoking Cessation Smartphone App for People With Serious Mental Illness

**DOI:** 10.2196/games.8881

**Published:** 2018-01-16

**Authors:** Roger Vilardaga, Javier Rizo, Emily Zeng, Julie A Kientz, Richard Ries, Chad Otis, Kayla Hernandez

**Affiliations:** ^1^ Center for Addiction Science and Technology Psychiatry and Behavioral Sciences Duke University Durham, NC United States; ^2^ Design Use Build Department of Human Centered Design and Engineering University of Washington Seattle, WA United States; ^3^ Department of Psychiatry and Behavioral Sciences University of Washington Seattle, WA United States; ^4^ Chad Otis Illustration & Design Seattle, WA United States

**Keywords:** smoking cessation, mHealth, serious mental illness, user-centered design, gamification, acceptance and commitment therapy

## Abstract

**Background:**

Smoking rates in the United States have been reduced in the past decades to 15% of the general population. However, up to 88% of people with psychiatric symptoms still smoke, leading to high rates of disease and mortality. Therefore, there is a great need to develop smoking cessation interventions that have adequate levels of usability and can reach this population.

**Objective:**

The objective of this study was to report the rationale, ideation, design, user research, and final specifications of a novel smoking cessation app for people with serious mental illness (SMI) that will be tested in a feasibility trial.

**Methods:**

We used a variety of user-centered design methods and materials to develop the tailored smoking cessation app. This included expert panel guidance, a set of design principles and theory-based smoking cessation content, development of personas and paper prototyping, usability testing of the app prototype, establishment of app’s core vision and design specification, and collaboration with a software development company.

**Results:**

We developed Learn to Quit, a smoking cessation app designed and tailored to individuals with SMI that incorporates the following: (1) evidence-based smoking cessation content from Acceptance and Commitment Therapy and US Clinical Practice Guidelines for smoking cessation aimed at providing skills for quitting while addressing mental health symptoms, (2) a set of behavioral principles to increase retention and comprehension of smoking cessation content, (3) a gamification component to encourage and sustain app engagement during a 14-day period, (4) an app structure and layout designed to minimize usability errors in people with SMI, and (5) a set of stories and visuals that communicate smoking cessation concepts and skills in simple terms.

**Conclusions:**

Despite its increasing importance, the design and development of mHealth technology is typically underreported, hampering scientific innovation. This report describes the systematic development of the first smoking cessation app tailored to people with SMI, a population with very high rates of nicotine addiction, and offers new design strategies to engage this population. mHealth developers in smoking cessation and related fields could benefit from a design strategy that capitalizes on the role visual engagement, storytelling, and the systematic application of behavior analytic principles to deliver evidence-based content.

## Introduction

### Background

Smoking rates in the United States have been reduced to 15% in the past decades [1]. However, this downward trend is not present in people with serious mental illness (SMI) [2]. This population, which is characterized by people with chronic mental health symptoms and functional impairments that interfere with major life activities, typically encompasses individuals with a diagnosis of schizophrenia, schizoaffective, bipolar, and recurrent major depression [3]. People with SMI have smoking rates of up to 88% [4-6], have high levels of disease [7], and lose 25 years of life expectancy [8].

Survey research indicates that this population has rates of adoption of mobile technology that range between 72% and 81% [9,10], closing the gap with the 95% adoption of the general population [11], and thus presenting an opportunity to develop mobile smoking cessation apps that address the treatment needs of this population.

Despite the increasing number of digital interventions developed for people with SMI [12-17], no mobile intervention for smoking cessation has been described in the scientific literature for this vulnerable patient population. This shortage of smoking cessation mHealth research for SMI is not surprising when considering the larger context of smoking cessation mHealth. There are over 540 smoking cessation apps in the market [18], but only 2 have been tested in randomized controlled trials [19,20], and to our knowledge, there are no reports of their user-centered design research.

This lack of reports on the user-centered design process of mHealth interventions for smoking cessation is problematic, because (1) the determination of the active therapeutic ingredients delivered by an app should be the result of a careful design process, (2) poorly designed software systems have an impact on their ultimate efficacy and when not usable can be a waste of resources [21,22], and (3) unreported design research undermines design reproducibility and our body of knowledge. Thus, user-centered design research of mHealth interventions is not only an important step to ensure their efficacy and usability, but also an important way to advance our scientific knowledge.

People with SMI can have very low levels of adherence to digital interventions [12,23], which calls for an user-centered design process that addresses a series of known usability barriers in this population, such as persistent and moderate-to-severe mental health symptoms [24], low levels of educational attainment [25], cognitive deficits [26,27], and poor fine motor skills [28].

### Prior Work

In previous research, we found a direct link between key demographic factors and engagement with SmartQuit, a smoking cessation app designed for the general population. Specifically, we demonstrated that experiencing mental health symptoms, being female, and having low levels of educational attainment predicted low levels of engagement with the app at a 3-month follow-up [29]. In subsequent research, we further identified specific usability barriers encountered by people with SMI when using NCI QuitPal, a smoking cessation app developed by the National Cancer Institute (NCI) [30]. Despite adherence to US Clinical Practice Guidelines (USCPG) for smoking cessation, our study directly showed that NCI QuitPal led to critical performance errors and low user experience among smokers with SMI, suggesting the need for new design approaches for this population.

### Goal of This Study

From these initial studies, we planned to develop Learn to Quit, a smoking cessation app tailored to this often neglected and vulnerable population. Consistent with the need to report the user-centered design process of mHealth interventions, the aim of this paper was to describe the rationale, ideation, prototyping, design, user research, and final feature set of Learn to Quit, a smoking cessation app tailored to individuals with SMI that will be subsequently tested in a randomized controlled feasibility trial (clinicaltrials.gov NCT03069482).

## Methods

**Figure 1:**
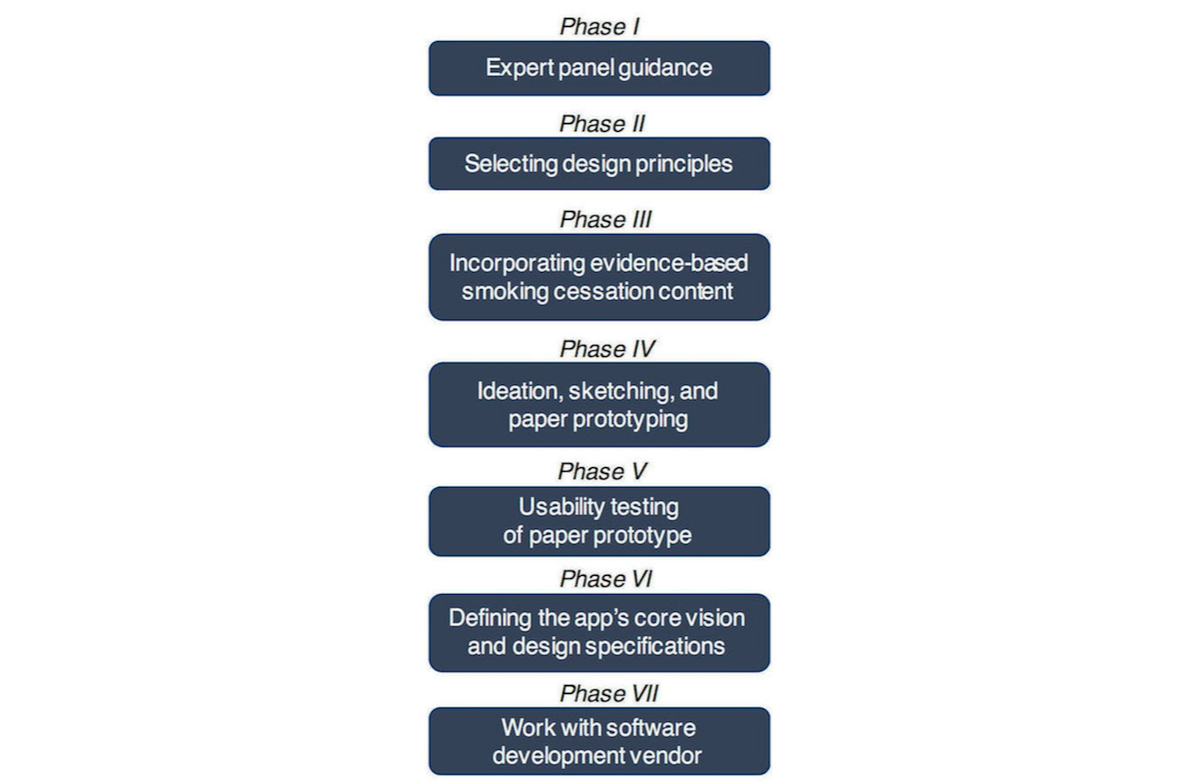
Methods and materials.

Our user-centered design methods and materials are summarized in Figure 1. This formative study was organized in 7 phases consistent with the user-centered design framework [31,32], which includes the following key activities: (1) understanding and specifying the context of use (phase I); (2) specifying the user and organizational requirements, such as the active ingredients of the behavior change intervention (phases II-III); (3) produce design solutions (phase IV); and (4) evaluate the design (phase V). Our design process also involved users throughout the design and development process, addressed the whole user experience (both usability and user experience), and incorporated multidisciplinary perspectives, which are key principles in user-centered design [31].

Phases VI and VII are not part of the user-centered design process per se, but are important steps in design implementation, which are also documented in the user-centered design literature [33]. Substantial progress in each phase was necessary to initiate meaningful progress in the following phase. However, at times, these phases overlapped with each other. For example, phase IV overlapped with phase V, because feedback from usability testing was used to modify or edit the original sketches and paper prototype. Conversely, completion of phase VI was a required step to initiate phases VI and VII. All study procedures were approved by the Institutional Review Board of the University of Washington.

### Phase I: Expert Panel

To better understand the needs of our target population, we sought to get the perspective of patients from this population and their providers. Expert guidance has been used in prior work to inform app development efforts [34]. We formed an expert panel composed of 2 social workers, 1 psychiatric case manager, 2 psychiatrists, and 2 smokers with SMI. After a project introduction, we addressed the panel with 2 research questions: (1) What are the biggest challenges for people with SMI to quit smoking? and (2) How could we design a highly engaging app for smokers with SMI? The first and second authors took notes from comments and observations, discussed them to identify agreements and focal points, and organized them for qualitative review. No formal thematic analysis of these interviews was conducted.

### Phase II: Selection of Design Principles

Before designing the app, we prespecified 2 sets of design principles: (1) general learning principles based on applied behavior analysis and (2) design principles specific to individuals with SMI. Applied behavior analysis is a scientific discipline focused on developing strategies and behavior modification techniques in areas of social relevance based on principles of learning. Use of these principles has shown promise for the treatment of addiction in people with SMI [35,36], and these principles have been used in the design of games and other health apps [37]. Design principles specific to individuals with SMI have typically addressed the cognitive deficits and mental health symptoms encountered by this population. This approach has been used in previous work designing technologies for people with SMI [38], and it is supported by the general literature with regard to the importance of adjusting designs systems to meet the cognitive model of the user [39-41].

### Phase III: Incorporating Evidence-Based Smoking Cessation Content

Smoking cessation app content was selected from behavior change interventions supported by the empirical literature (eg, clinical trials) and from process research suggesting a theoretical link between intervention components and the symptoms typically experienced by our target population (see below “Evidence-Based Smoking Cessation Content” subheading in the Results section). This process ensured the theoretical grounding of the app and its evidence-based foundation.

### Phase IV: Ideation, Sketching, and Paper Prototyping

We used several user-centered design tools to ideate a prototype of the app. This included (1) the creation of personas, a technique aimed at increasing the designer’s emotional understanding of the end user by creating a short narrative of their motivations, context, and personal characteristics [42-44], and (2) sketching and paper prototyping, an important component of the design process consisting the use of paper drawings to quickly iterate on variations of app structure, app interactions, and the layout of content [42,45].

### Phase V: Usability Testing of Paper Prototype

#### Procedures

Sketches and images developed during the ideation and paper prototyping phase provided the basis for usability testing. To simulate the app experience, we used app prototyping software (POP, Marvel, London, UK). The software was installed on iPod Touch devices, and the prototype was presented to smokers with SMI during a single 45 min session. Our key inclusion criteria were as follows: (1) being an adult who smokes at least 5 cigarettes per day, (2) receiving outpatient mental health treatment and medication by a psychiatric provider, and (3) being fluent in

Usability testing procedures included (1) completing a series of tasks with the simulated app, (2) evaluation of user experience with semistructured interviews, and (3) rating the prototype using the system usability scale (SUS) [46]. Preceding our user interviews with a series of hands-on tasks provided the user with a more in-depth experience with the app prototype, and therefore allowed us to gather more meaningful and concrete feedback from users. Finally, our global assessment of usability, the SUS, was conducted at the very end to give users an opportunity to summarize their feedback. Given the iterative nature of this testing phase, we modified app design features after each participant and provided the new version to the following participant. This iterative process is standard in formative evaluations [47].

**Figure 2:**
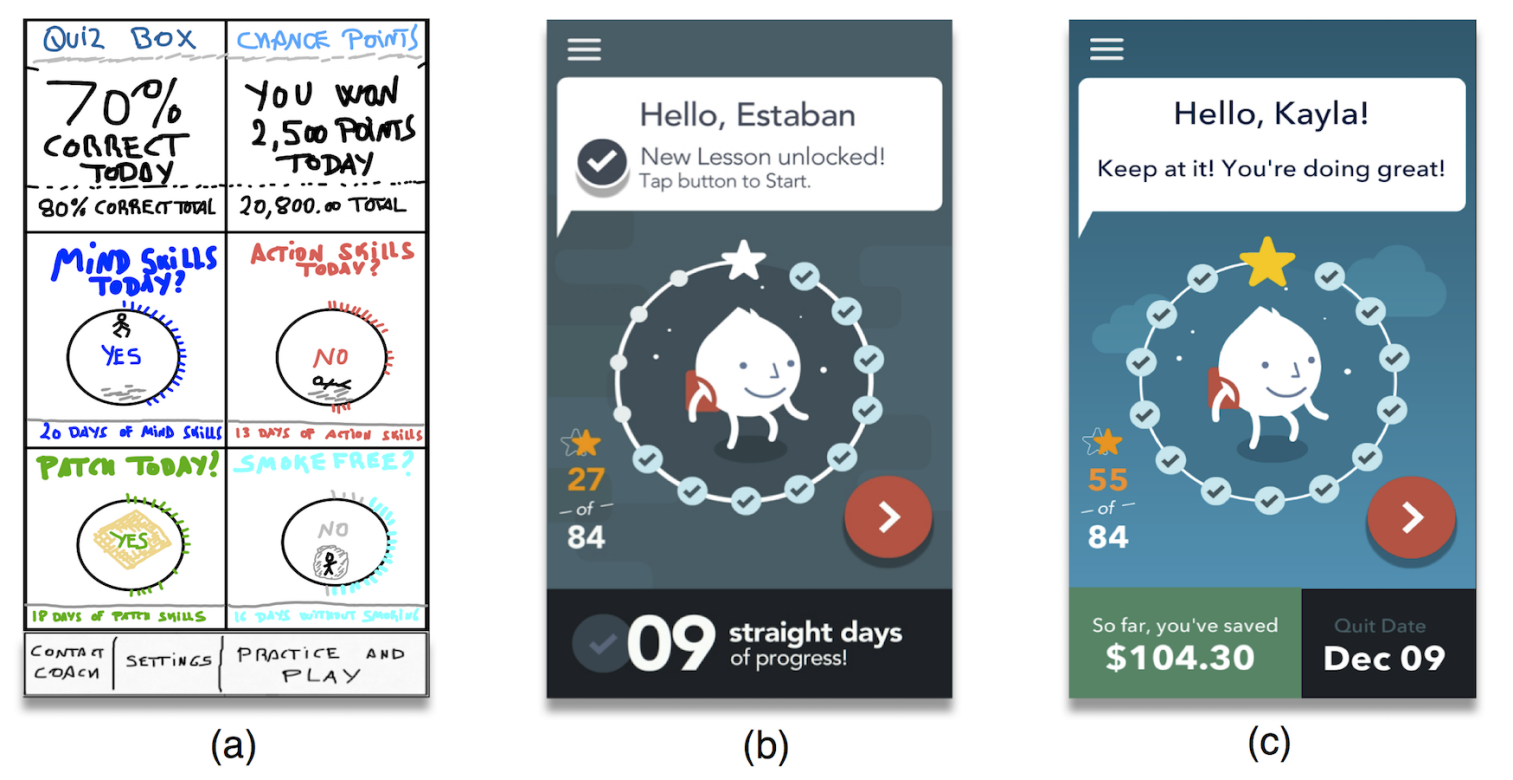
These wireframes represent how our app’s initial Home Screen evolved throughout our design process. From left to right: (a) Home Screen sketch, (b) pre-14 Home Screen, and (c) post-14 Home Screen. Wireframes (b) and (c) are examples of the 2 types of Home Screen status: dark green, indicating that the user is still completing the 14 modules of Learn to Quit, and light blue, indicating that the user already completed the Learn to Quit lessons and is ready for his first quit attempt.

**Figure 3:**
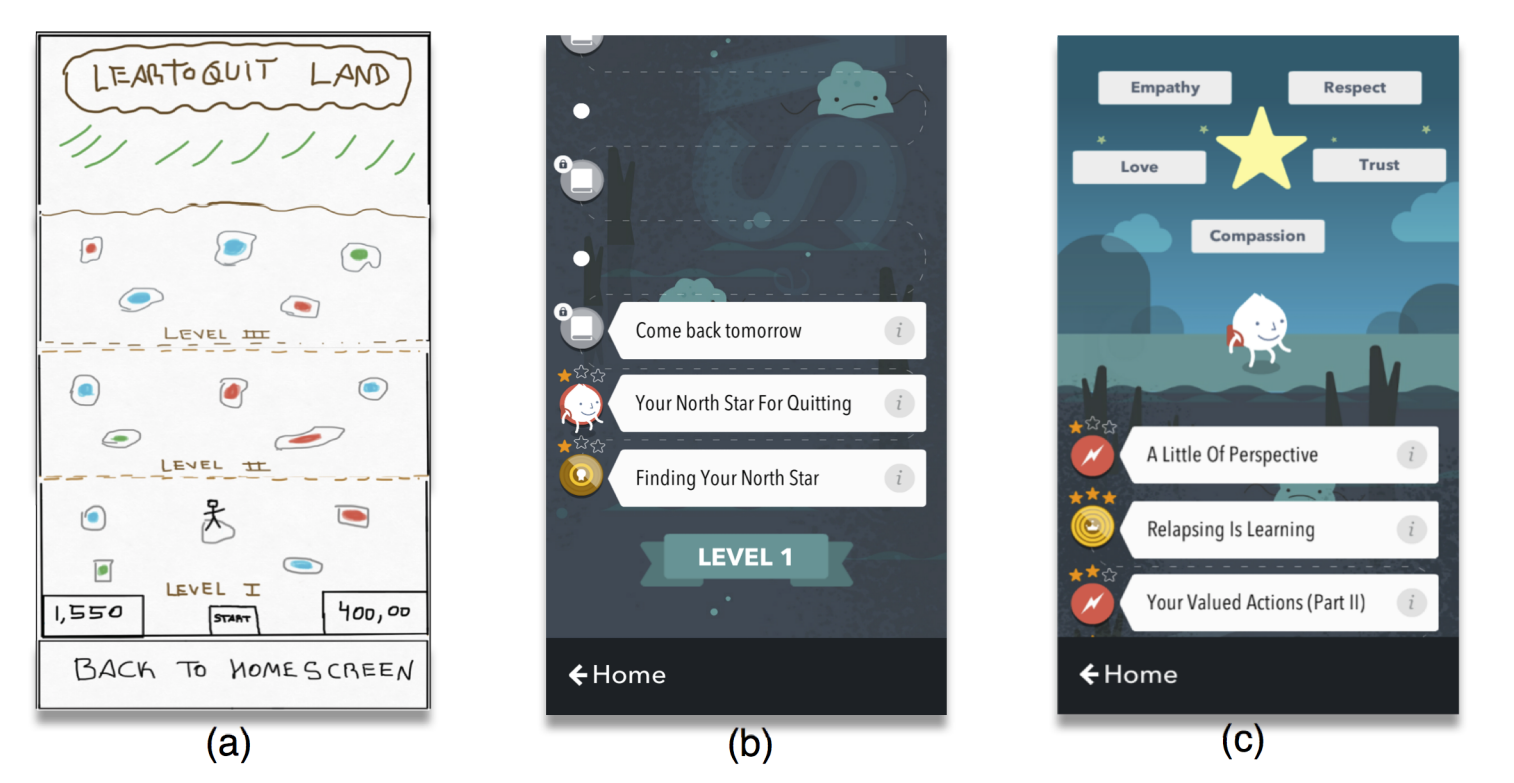
These wireframes represent how our app’s initial Play Screen evolved throughout our design process. From left to right: (a) Play Screen sketch, (b) pre-14 journey map, and (c) post-14 journey map. Wireframe (a) presents a character that needs to “jump” from stone to stone to “pick up” skills for quitting while navigating through a “swamp of urges.” In wireframe (b), the user has completed the “Finding Your North Star” lesson and practiced the “Your North Star for Quitting” skills module. Wireframe (c) presents a user who has completed all levels of the Learn to Quit journey and motivated by his values for quitting has metaphorically reached “Learn to Quit Land”.

Our usability testing tasks evaluated the following elements of the app prototype: (1) an introductory tutorial, (2) overall Home Screen navigation (Figure 2, panel a), (3) overall Play Screen navigation (Figure 3, panel a), (4) access to technical coach feature (Figure 2, panel a), and (5) access to learning score and practice scores (Figure 2, panel a). Consistent with usability testing guidelines [47], our procedures reminded the interviewer to keep neutrality in response to user comments and behavior, read the script verbatim to each user, and observe and keep track of user comments and behavior. Finally, for the purposes of this study, we did not conduct observational coding of users’ behavior during task completion. Instead, we relied on the qualitative analysis of user feedback and the SUS.

#### Measures and Analysis

The SUS is a valid and reliable 10-item 5-point Likert scale with scores that range from 0 to 100. Higher scores indicate higher levels of usability, with scores above 68 indicating above-average usability [46]. The SUS can be further analyzed based on 2 subscales that measure usability and learnability of the software system. Learnability refers to the user’s level of ease in gaining proficiency with a software system. These subscales are interpreted following the same range and direction of the overall scale [48]. Semistructured interviews were recorded, transcribed, and analyzed using thematic analysis, an inductive qualitative method that organizes verbal content based on similarity, dependence, and proximity to identify key themes and opportunities for innovation [49-51].

### Phase VI: Defining of the App’s Core Vision and Design Specifications

We created a design specifications document that laid out the app’s overall vision, look and feel of the interface, and its basic components and features [33]. This document was used to facilitate communication with a software development company.

### Phase VII: Work With a Software Development Vendor

In this final phase, we worked with a software vendor to materialize the vision and design specifications that resulted from our formative study.

## Results

In this section, we describe the results of our formative study by focusing on a summary of our expert panel feedback, describing an initial paper prototype of the app and the results of its usability testing. This formative study resulted in a clearer definition of the app’s core vision and helped define the scope of work to be conducted by a software development company. Results from the remaining user-centered design processes and materials (ie, design principles, theory-based content, paper prototyping) are presented throughout a final section that lays out the app’s final characteristics and features and how they were informed by those processes and materials.

### Results From Our Formative Study

#### Expert Panel Results

The panel emphasized the specific challenges faced by this population when trying to quit (Table 1). This included the panel members’ view that, as opposed to the general population, patients with SMI are more concerned about ongoing medical and mental health challenges (eg, metabolic syndrome, suicidality) rather than dying from cancer. Although not consistent with the behavioral economics literature [52], in the panel members’ experience as providers, motivations to quit in people with SMI are either different or less pronounced than in the general population. To add to this challenge, providers indicated that both withdrawal symptoms and ongoing mental health symptoms were a common concern in their patients when discussing the possibility of quitting smoking (eg, triggering a psychotic event). Needing preparation to quit was also emphasized, arguing that patients would prefer a system that teaches them a set of skills for quitting, rather than a system that emphasizes setting up a quit date and monitoring maintenance.

To solve this lack of motivation and concerns about the process of quitting, the panel outlined a series of strategies that could engage the users with this app. One of them was the use of meaningful images and storytelling. Some level of gamification was viewed as important to engage users with SMI. In addition, the panel argued that as a way to compensate for the overwhelming task of quitting smoking, the system should include progressive disclosures, make sure it rewards small victories, and add external motivation (eg, money saved). Social networking and monitoring of medication aids were also mentioned. Finally, a few other themes emerged during our discussion, including challenges related to the use of technology in this population, the social context of these patients (eg, risk of having their smartphone being stolen), and the benefits of personalization and provider support during the initial stages of nicotine withdrawal and beyond.

#### Initial Results From Ideation, Sketching, and App Prototyping

On the basis of input from our expert panel and the authors’ experience as clinical providers, we created 3 personas: (1) *Esteban*, a 55-year-old male with schizophrenia, 35 years of smoking history, psychiatrically stable, and with a range of medical complications; (2) *Martin*, a 31-year-old male with a diagnosis of bipolar disorder, who is a light smoker, and holds a stable job; and (3) *Julia*, a 43-year-old female with recurrent major depression and a history of drug use, who is taking care of a young daughter. These personas guided the design process and helped ensure that the initial prototype was responsive to the physical and emotional contexts of a broad spectrum of people with SMI, from psychotic to chronic affective disorders (see Multimedia Appendix 1).

**Figure 4:**
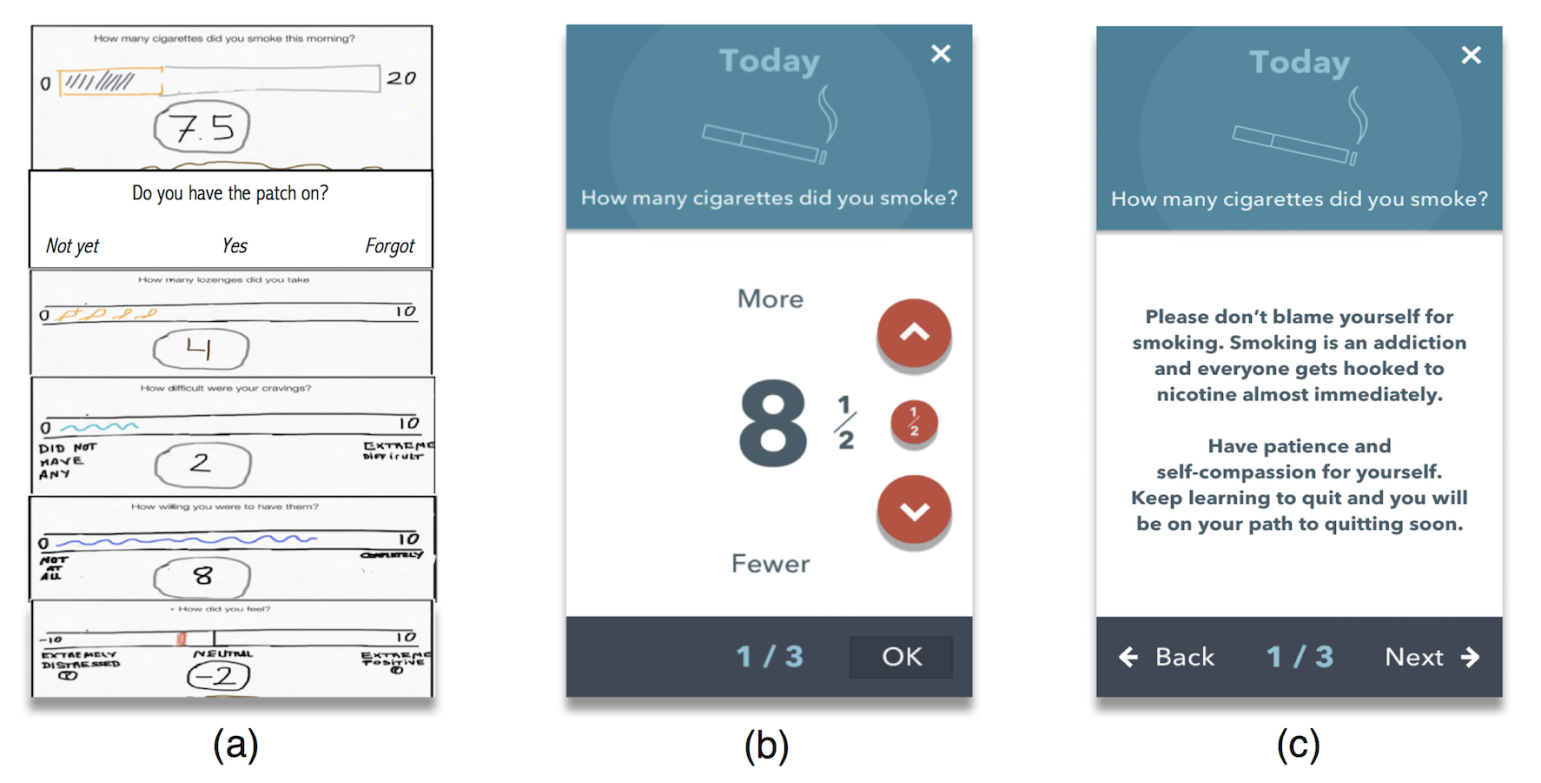
These wireframes represent how our initial tracking feature evolved. From left to right: (a) tracking feature sketch, (b) cigarette tracking, and (c) personalized cigarette use feedback. As opposed to wireframe (a), in which we planned to use a single wireframe to collect all desirable tracking dimensions, in the final app, we used separate wireframes for each dimension (eg, smoking, mood). Note in (b) that users could report smoking half cigarette. Wireframe (c) is an example of personalized feedback following a user who reported smoking between 5 and 10 cigarettes.

**Figure 5:**
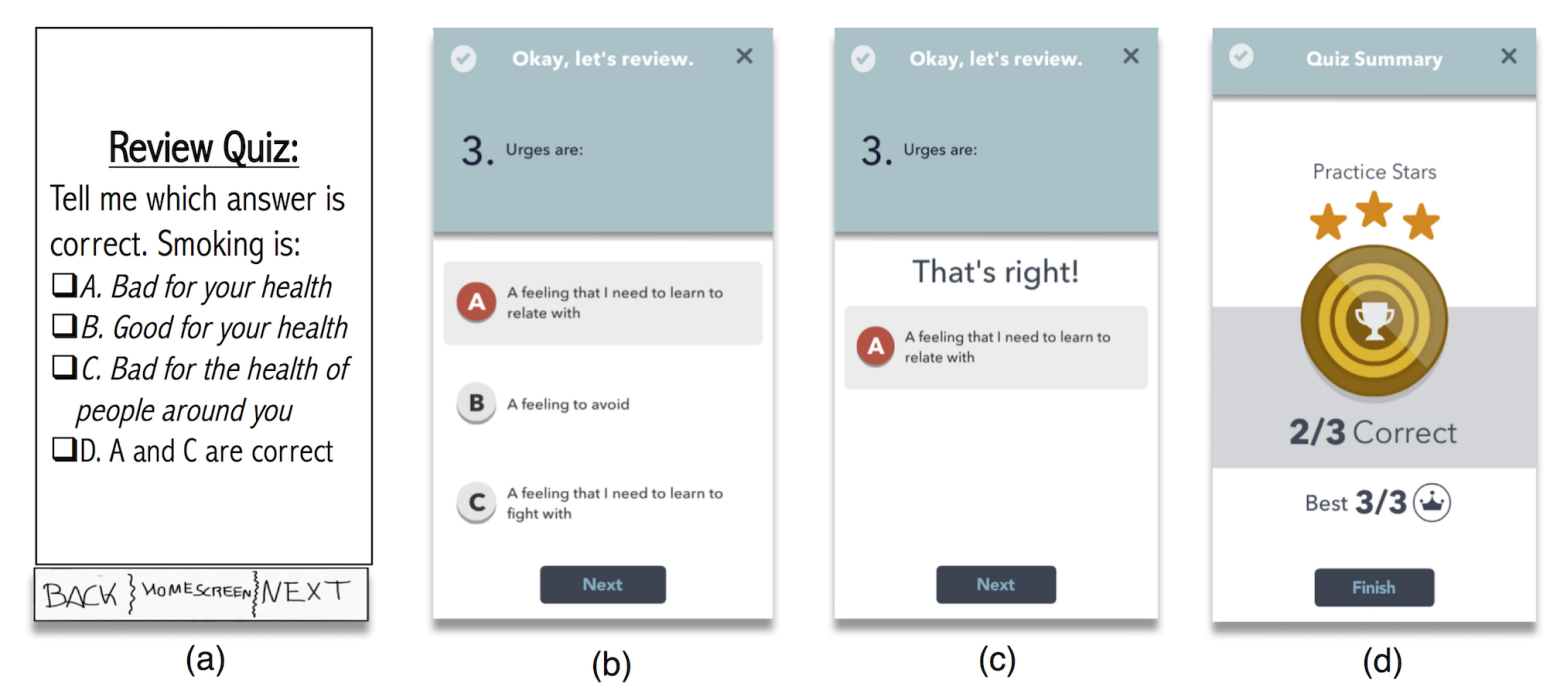
Wireframe examples of an initial sketch of a “Review Quiz” and a final Learning Module Quiz. Quizzes were presented at the end of the learning modules, and contained 3 questions each. From left to right: (a) Review Quiz sketch, (b) example of question for the “Key to Quitting” module, (c) feedback to correct answer that is followed by game reward sound, and (d) summary of quiz results, which indicates number of correct answers, best answer of all times, and number of practice stars gained (1 for each practice with a total of 3 per module).

**Figure 6:**
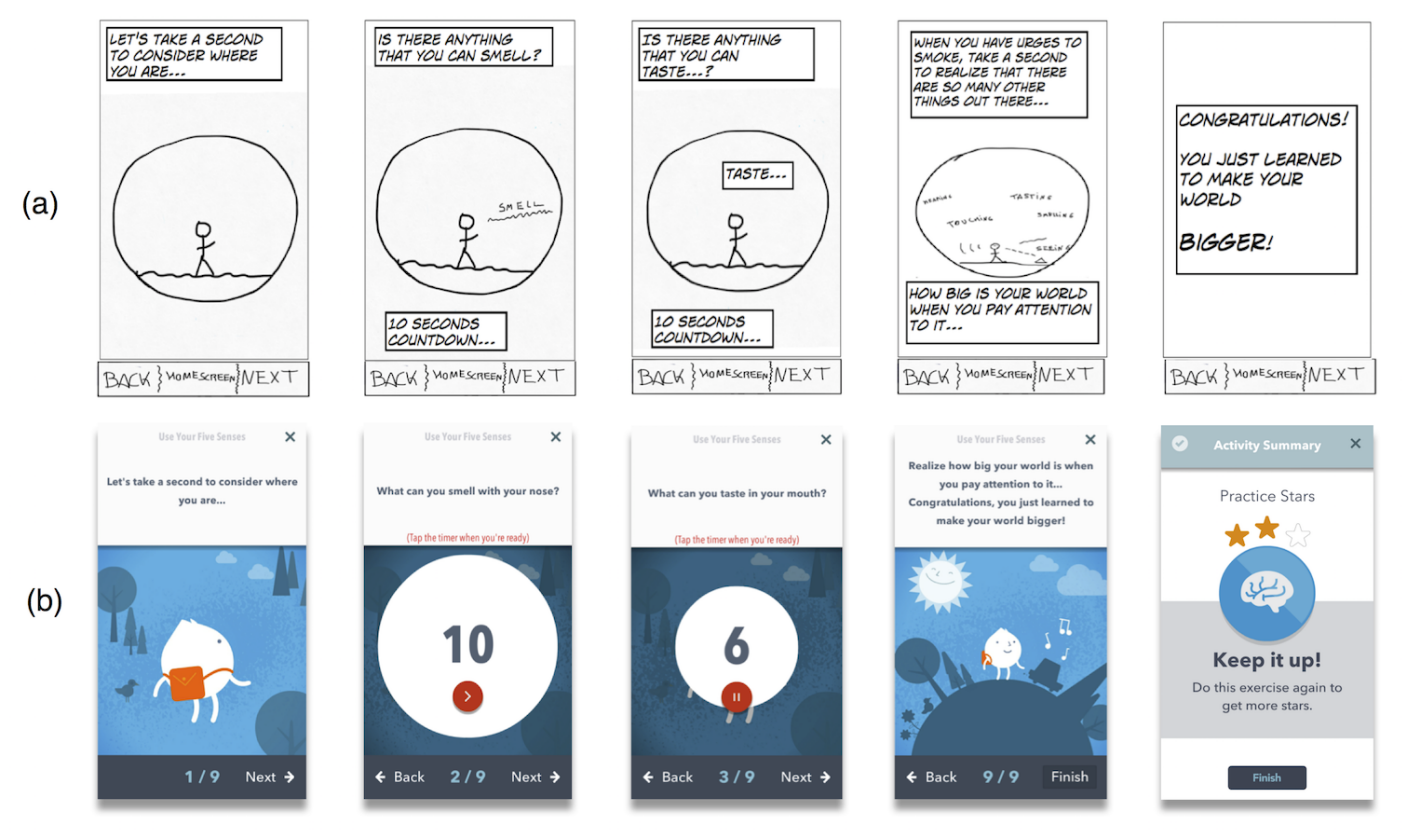
Selection of wireframes of a smoking cessation skill (ie, Use Your Five Senses) designed to encourage self-awareness of our 5 senses. From top to bottom: (a) sketch of Use Your Five Senses skill and (b) final Use Your Five Senses skill module. Wireframes in panel (b) include five 10-second timers to assist the user focus their attention. They provide visual and tactile cues to mark the end of each practice of focused attention.

These personas were used as inspiration to sketch our first wireframes (ie, sets of images displaying the functional elements of a website or app) and the overall app prototype, which was limited to a few basic components: A Home Screen (see Figure 2, panel a); a Play Screen (see Figure 3, panel a); a Tracking Screen (see Figure 4, panel a); a Quiz Screen (see Figure 5, panel a); a proof-of-concept smoking cessation module (see Figure 6, panel a); and an onboarding tutorial. To minimize cognitive demand in people with SMI, the app had only a few buttons (eg, “back,” “next,” and “Home”). These buttons were large, an important feature given the fine motor deficits observed in people with SMI [28] and findings from previous research in this population [30,38].

**Table 1:** Expert panel themes. Each of the insights of our expert panel are organized by a question and accompanied by a short description.

Questions and themes		Description
**Question 1: What are the biggest challenges for people with serious mental illness to quit smoking?**		
	Motivation to quit	Life expectancy is not generally a motivation to quit in this population
	Quitting without preparation	Early attempts to quit without enough preparation, and/or lacking a step-down quitting process
	Withdrawal symptoms	Fear of experiencing withdrawal symptoms days after quitting
	Mental health symptoms	Ongoing anxiety, depression, stress, and psychotic symptoms during the quitting process
**Question 2: How could we design a highly engaging app for smokers with serious mental illness?**		
	Meaningful visuals	The ability to display pictures of inspiring objects, people, sites, or pets
	Having a “video game” feel	The appeal of video games or “game like” features (eg, “bingo”)
	Social networking	The possibility of sharing with peers
	Storytelling	The use of interactive characters (eg, dog) for storytelling
	Encouraging activation	The importance of increasing activation (eg, exercise) to facilitate quitting and cope with withdrawal
	Progressive disclosure	Unlocking bits and pieces of the app, such as a picture or a message, as the user makes progress
	Rewarding small victories	Reinforcing small victories toward quitting (eg, one day without smoking)
	Money savings motivation	Small money savings (eg, a few extra dollars a week)
	Medication aids	Medication education and a system to help patients adhere to medication intake
**Miscellaneous themes**		
	Technological literacy	Lack of smartphone knowledge was viewed as a potential barrier to app use
	Predominant use of Android	The need to build an app in Android was viewed as important to secure access in this population
	Provider check-ins	Flexible check-ins (eg, text messages) were emphasized to enhance engagement with the app
	Need for personalization	An app that was customizable to each patient was deemed as important
	Stealing	Concern that some patients with serious mental illness might have their devices stolen

**Table 2:** Key baseline features of usability testing subjects and corresponding system usability scale (SUS) scores. Scores above the usability standard cut-off (>68) are indicated in italics.

Participant number	Mental health treatment	Years in mental health (mean=25)	Years smoking (mean=20)	Cigarettes per day (mean=11)	SUS usability (mean= *80*)	SUS learnability (mean=60)	SUS total (mean= *74*)
P1	Case manager, psychiatric nurse	25	9	13	*72*	*100*	*78*
P2	Case manager, psychiatric nurse	29	10	7	*88*	*75*	*85*
P3	Case manager, psychiatrist	20	32	10	44	25	40
P4	Case manager, psychiatrist	39	35	15	*81*	50	*75*
P5	Case manager, psychiatrist	12	14	10	*94*	*88*	*93*

#### Usability Testing Results

A total of 5 daily smokers recruited from an outpatient mental health clinic participated in usability testing of the app prototype. They averaged 44 years of age (standard deviation [SD] 7.5), and the majority were female (4/5) and had less than a college education (4/5). Of the 5 participants, 1 was multiracial, 1 African American, and the rest were white. Our sample group smoked an average of 11 cigarettes per day (SD 3), and most had an extended smoking history (mean 20 years, SD 13). Although we did not conduct diagnostic interviews, all participants were patients from a community mental health clinic, had an assigned psychiatric case manager and a psychiatric provider, were currently taking psychiatric medication, and on average had received mental health treatment for 25 years (SD 10). See Table 2 for a breakdown of individual baseline characteristics.

##### System Usability Scale

Overall, participants’ levels of usability with the prototype were above the standard cutoff, suggesting that the initial prototype had promise. This was reflected in both the overall scale and the usability subscale. Conversely, the learnability subscale of the SUS did not reach the standard cutoff, although it reached high levels for 3 out of 5 individuals (see Table 2).

##### Thematic Analysis

Table 2 summarizes the results of the thematic analysis. To facilitate comparison with user testing of a nontailored app for smoking cessation in the same population (NCI QuitPal), we matched some of the themes resulting from our analysis with a previous usability testing study we conducted in smokers with SMI [30].

##### Key Learnings From Usability Testing of the Learn to Quit Prototype

Key learnings from usability testing can be organized in 3 areas: support for app design features, critical usability errors, and minor usability errors. First, we found that the following app design features improved the apps usability: (1) the prototype’s reduced number of app layers, (2) removing the need to use a keypad to enter and save information in the app, and (3) overall prototype’s simplicity. Although Learn to Quit’s simple app structure and navigation features were the result of a previous user-centered design study we conducted in the same population [30], this study allowed us to test a specific and concrete solution to those previously identified usability issues. User experience more directly confirmed that participants appreciated the simplicity of the design and the use of cartoons and gamification, and had a positive response to our paper prototype of a smoking cessation skill (see Tables 3 and 4).

Second, usability testing identified a critical usability error with our paper prototype. Specifically, it revealed that our original Home Screen was confusing to most users (see Figure 2, panel a). Users had difficulty in understanding the Home Screen structure and the purpose of each Home Screen subpanel. This is reflected in the prototype’s suboptimal SUS learnability score.

Finally, we identified minor usability problems with the prototype, including small font in the subpanels and confusion about specific language. These usability errors led to a final Home Screen that had a simpler layout and removed most of its original displays and content (see Figure 2, panels b and c).

**Table 3:** Usability testing results for Learn to Quit prototype (n=5) matched with comparable usability testing results from a previous user-centered design study (n=5) we conducted in a smoking cessation app designed for the general population (QuitPal) [30].

Theme	Quote/Observation/Feature	
	Smoking cessation app (QuitPal); (SUS^a^=65.5)	Learn to Quit paper prototype; (SUS= *74*)
Difficulty entering information in the app	Unable to “pull up the keypad”	The need to use the keypad was removed from the prototype
Difficulty saving information	Failure to identify and press “save” button at the top of screen	The need to use a “save” button was removed from the prototype
Getting lost in app layers	“It took me a long time to get back to that menu frame”	No observed confusion about how to return to the Home Screen
Tremor and fine motor skills	“[buttons were] too close together”	P5^b^: “I like how the letters are big”

^a^SUS: System Usability Scale; scores above the usability standard cut-off (>68) are indicated in italics.

^b^P: Participant.

**Table 4:** Themes identified during usability testing of the Learn to Quit app prototype (n=5).

Themes	Representative quote^a^
Interested in gamification of smoking cessation skills	P1: “That would be so cool! A point every day” P2: “[the most exciting] the points” P4: “this would be kind of fun”; “So this is like a little game and people often play a lot of games” P5: “That’s a good thing […] that builds confidence”
Drawn by cartoons and storytelling	P3: “The cartoons, the whole thing. It’s got great spirit” P4: “that’s very cute!”; “I like it, it’s cartoony-like”; “it is very eye-catching” P5: “So you got a cartoon character! That’s what I was thinking. It was right on. That works for me”; “Mm, cartoon characters, yes!”
Appreciating simplicity	P2: “It was simple, informative, easy to use” P4: “[I like it]...when you’re a kid and you’re learning something new, it’s basic and it’s not all overstimulated, to put it that way…” P5: “It just goes right in your mind”; “this looks really simple too and it looks good”
Proof of concept: Acceptance and Commitment Therapy module showed promise	P1: “I wish you guys could send it to me so that I could practice it and learn it” P4: ”it’s like this too shall pass […], makes sense.”; “Mind and action skills. That’s pretty good. Because it’s like when you go to an AA” P5: “It relaxed me”
Home Screen confusion	P1: “It’s very small and I can’t see what it is.” P2: “I don’t know if that’s an “I” or not.” P3: “The first part of the app is so busy…”

^a^P: Participant.

#### Design Specifications and the App’s Core Vision

On the basis of this formative study, we created a technical document laying out the app’s structure and its core screens and features. We named the app Learn to Quit and synthesized the app’s core vision with the following: learn, practice, and play. Learning referred to the process of being exposed to daily modules that explain different smoking cessation concepts. Brief quizzes would help the user retain and learn those materials. Practice referred to the actual practice of smoking cessation skills in the form of brief daily exercises. Practice should lead to “mastery” of the learned materials. Play referred to the user’s opportunity to participate in a game comprising completing learning and practice modules and earning rewards along the way. The role of play was to promote higher levels of app engagement and commitment to learn.

#### Software Development Timeline

In May 2015, we filed a Report of Innovation at the University of Washington (ROI#47274) with the design specifications document of the app and proceeded to approach a company to develop a software-coded version of the app. Learn to Quit was built between August 2015 and October 2015. Smashing Ideas Inc. [53], a design and development agency, contributed to the refining and enhancement of the app prototype.

### Learn to Quit’s Core Features

#### Evidence-Based Smoking Cessation Content

Learn to Quit’s main active ingredient is Acceptance and Commitment Therapy (ACT) [54]. We chose this behavior change approach because (1) it has promising results across multiple smoking cessation clinical trials [19,55-58]; (2) its components have shown to predict smoking cessation outcomes [59-61]; (3) it is an intervention originally developed to provides skills to cope with mental health symptoms [54]; and (4) it has been successfully adapted to people with SMI [62-65]. These 4 characteristics made ACT a suitable evidence-based approach for smoking cessation in this specific population.

ACT has 3 components relevant to smoking cessation: *awareness,* or the smoker’s ability to recognize smoking triggers and urges; *openness*, or the smoker’s willingness to experience smoking urges and triggers; and *values activation*, or the smoker’s active engagement in values-based activities related to health. The app content was therefore designed to deliver techniques related to each of those components, which predicted smoking cessation outcomes in previous research [59,60]. A secondary active ingredient of our intervention was adherence to key elements of USCPG for smoking cessation [66]. These guidelines include setting up a quit date, proper use of medication aids (eg, nicotine patch), and preparing for relapse. These 2 active ingredients (ACT and USCPG) were integrated to ensure an evidence-based design approach to app development.

We adapted ACT to a mobile format by creating 14 modules of ACT+USCPG content and 14 modules of exercises to practice smoking cessation skills (see Multimedia Appendix 2). We chose a 14-day program that would be consistent with USCPG, which recommends 2 weeks of preparation for quitting, and would balance the need for gradual exposure to smoking cessation content while providing a concrete timeline. Each module presented a short narrative that exemplified ACT+USCPG content and explained it from the perspective of someone with nicotine addiction and SMI. Each concept was presented in a variety of narratives and exercises to increase learning generalizability (see principle of multiple exemplar training in Multimedia Appendix 3). In addition, each lesson ended with a quiz designed to further enhance comprehension and retention (see Figure 5, panels b and c). We arranged these modules so that completing a lesson per day was necessary to unlock new lessons the next day (see principle of differential reinforcement of successive approximations in Multimedia Appendix 3).

Completion of skills modules was optional to increase the user’s perceived behavioral control [67]. ACT+USCPG content was developed and organized so that it would gradually increase in complexity as the user advances through each of the 3 levels (see section below on app gamification).

#### Simple Screens, Large Buttons, and a Predictable App Structure

To address the cognitive deficits observed in this population, we incorporated feedback from our formative study and followed recommendations from previous literature [30,38-41,68]. See Multimedia Appendix 3 for a list of key design principles we used to address SMI in our app. The result was an app with simple screens, large buttons, and a simple structure. The Home Screen (Figure 2, panels b and c) only included 2 buttons, 1 to access the Play Screen, which unlocked the module content described above, and 1 button to access the app settings. Usability testing suggested that the Home Screen should incorporate only a few elements. Thus, the Home Screen had as its focus a character surrounded by a circle of checkmarks, indicating overall progress in modules’ completion.

The Play Screen (Figure 3, panels b and c) represents a game in which each day the user takes a step forward toward completing a smoking cessation module. This screen had a linear structure, with older modules at the bottom and newer modules toward the top. Each module ranged between 6 and 24 screens, all of which had a reduced amount of semantic information, a simple color palette, consistent font size and type, and a predictable structure. All remaining wireframes in the app were presented in the form of cartoon scripts divided in 2 panels: the upper panel included the text and the lower panel a complementary image. We made each panel identical in size to maximize consistency and minimize cognitive load. This format was consistent with our panel’s emphasis on the use of storytelling and interactive characters to engage patients with SMI. We also avoided the use of videos and audio. We hypothesized that the use of sliding cartoons and vignettes could maximize retention and comprehension of smoking cessation skills, as this format provides the user with more control over the speed of presentation of smoking cessation content and the ability to stop or review a single vignette for as long as needed.

#### Gamification of Smoking Cessation Content

Consistent with our expert panel’s feedback, usability testing, and the empirical literature on the use of games not for entertainment, such as health or education (ie, serious games) [67,69], we designed an app that gamifies smoking cessation content. The main concept was designing a game in which the smoker metaphorically overcomes a swamp of urges and learns quitting skills along the way (see Figure 3). Gamification included interactive quizzes that provide immediate feedback about quiz results (see Figure 6, panel d), a component that has been correlated with long-term improvements in eHealth interventions [70]. Feedback included auditory cues for right or wrong answers and a reward system consisting of checkmarks, badges, cups, and crowns (see Applied Behavior Analysis section below). Repeated practice was encouraged with a reward system that provided stars every time a module was completed. This star system was consistent with the narrative of the app game (ie, module “Finding Your North Star”; see Multimedia Appendix 2). Sliding through module screens was also followed by video game sounds and feedback. We also added interactive elements to our tracking feature. Specifically, a 3-tiered system of personalized feedback was incorporated to diminish assessment burden. That is, we categorized all possible answers to a specific question (“How many cigarettes you smoked today?”) into 3 different levels and we created a custom message for each, acknowledging the users’ answer and encouragement to move forward in their journey (eg, smoking less than 5 cigarettes; see Figure 4, panels b and c).

#### Application of Principles of Applied Behavior Analysis

Behavioral principles (see Multimedia Appendix 3) informed the organization and delivery of smoking cessation content. These principles had the goals of (1) increasing retention, comprehension, and mastery of app content and (2) minimizing the impact of the cognitive deficits and low educational attainment of our target population [26,27,71]. For example, instead of providing the user with all smoking cessation content at once, we used the principle of successive approximations [72] to lay out increasingly complex ACT content throughout a 14-day period (see Multimedia Appendix 2 for a list of all the lessons and skills). Then, we applied the principle of multiple exemplar training [73]. Consistent with this principle, we presented multiple examples of a given concept or skill throughout multiple modules as a means to foster skills generalization to real-world settings.

In addition, the app used a combination of antecedent and consequential control strategies [72,74]. On the one hand, antecedent control was used in the form of notifications and Home Screen messages to prompt the individual to complete certain modules or tasks (Figure 2, panel b: “New Lesson unlocked! Tap button to Start”). On the other hand, consequential control was used by applying positive reinforcement arranged on a fixed ratio schedule [72]. More specifically, obtaining a new star was made contingent upon completing a module. This reinforcement ratio was used to encourage users to complete those modules at least 3 times (ie, a maximum of 3 stars could be obtained per module; see Figure 3, panels b and c), which could lead to a total of 84 stars (see Figure 2, panels b and c). Additional modules could be completed at any time but it was not incentivized with more stars.

A rewards scheme was also implemented to increase the reinforcing effect of responding correctly to lesson quizzes (see Figure 5, panel d). For example, an individual who gave 3 correct answers to 3 questions would receive a “crown.” Badges and cups were provided to users who responded 2 and 1 correct answers, respectively. Checkmarks were offered to users who completed the quiz but did not have any correct answer. Finally, we used negative reinforcement to promote daily completion of app modules. Specifically, the number of days in a row in which the user completed a module was indicated with a big bold number at the bottom of the Home Screen (see Figure 2, panel b). Not completing a new module one day was penalized with starting the count again from zero.

#### Emphasis in Visual Engagement and Storytelling

Because our expert panel and usability testing strongly supported the use of simple cartoons and visual storytelling, we created a gender-neutral character that rotated across the different stories and metaphors presented in each module (see Figures 2,3, and 6). The character enacts a variety of scenarios with the aim of exemplifying the experience of nicotine addiction and the smoking cessation skills offered in the app. Combination of imagery and text has been recommended in previous literature on mHealth in SMI populations [14]. The stories were told in very short sentences designed to avoid cognitive overload and maximize module completion. The purpose of these images and stories is to evoke an emotional connection and increase retention and comprehension of app content. For example, the Home Screen had 2 types of background: (1) dark green (Figure 2, panel b), to represent that the user is still completing the Learn to Quit journey and thus navigating through a “swamp of urges,” and (2) light blue (Figure 2, panel c), to indicate that the user has reached “Learn to Quit land” and thus is ready to quit. Similarly, the background of each module used a consistent and muted color scheme that maps into the 3 dimensions of the app’s theory-based content: (1) gray for Learning Modules, (2) blue for Mind Skills, and (3) red for Action Skills. The function of this color scheme was to offer a simple and predictable layout that would be more likely to fit the cognitive model of the user [40,41]. Previous research in people with SMI also indicated that bright colors can be overwhelming in this population, thus supporting our use of muted colors and a simplified color scheme [38].

#### Access to Technical Coaching

Our expert panel indicated that “technological illiteracy” was common in patients with SMI (Table 1). In the panel members’ opinion, many of their patients have never used a smartphone device, and therefore using successfully an app for smoking cessation could be a challenge. Furthermore, they commented on the complex social context of these patients and the potential need for more intense personal support. In our previous study testing a smoking cessation app in a small sample of this population [30], we observed some of these challenges, but we also noted that there was a wide range of technological literacy in this population, with some patients owning their own device and comfortably using smartphone apps. Therefore, we incorporated a simple technical coaching feature in our design that would serve as an aid to those that needed guidance, whereas at the same time maintaining the core vision of the app as a stand-alone intervention. This technical coaching feature consisted of a button in the settings section that allowed the user to call a preassigned technical coach. Eventually, this technical coaching role could be performed by addiction counselors or case managers and therefore serve to integrate the app within ongoing health care at a community mental health clinic or larger health care organization.

## Discussion

### Principal Findings

This paper reports the rationale, ideation, design, user research, and final features of a novel smoking cessation app developed for people with SMI, a population in great need of novel smoking cessation treatment. Building this app involved a user-centered design process that carefully considered a series of design principles to maximize comprehension and retention of smoking cessation concepts, minimize the impact of known challenges in people with SMI, and ensure the effective delivery of evidence-based smoking cessation content.

Results from our user-centered design process informed the features included in the final app. First, informed by our formative study and as suggested by the literature [14], we gradually delivered evidence-based smoking cessation content using imagery and simple semantic content. The primary active ingredient of this evidence-based content was ACT, which is an intervention that has empirical support as a smoking cessation intervention [55-58] and as an intervention to treat individuals with SMI [62-65]. This intervention was further integrated with smoking cessation recommendations from USCPG [66] to ensure alignment with best clinical practices.

Second, as suggested by the literature [30,38-41,68] and our user testing of a paper prototype, we created an app with very simple screens, buttons, and a predictable app structure that led to promising usability and user experience results. The paper prototype we created used a minimal set of app buttons and a very simple app structure, which probably contributed to scores above the usability standard cut-off. Our thematic analysis of user experience interviews was in line with these usability scores and further reinforced the inclusion of our final set of app’s core features.

Third, input from a panel of experts in SMI led to the idea of incorporating app gamification, visual engagement, and storytelling [67,69,75]. Use of these elements is consistent with the serious games literature for smoking cessation published in this journal [67]. More specifically, the app offered a module to help users identify their core values for quitting and used these values as the game’s objective (ie, module “Your North Star for Quitting”), which increases the user’s perceived behavioral control and intrinsic motivation [67]. In addition, it offered a clearly structured game that had functional utility [67], that is, successfully completing the game involved being exposed to a series of content known to help people quit smoking.

Fourth, we implemented a number of applied behavior analysis principles to maximize retention and comprehension of app content [37,76]. This included the use of progressive disclosures of smoking cessation content, variation and repetition of that content, a reward system, and modules that gradually portrayed complex smoking cessation concepts (eg, psychological awareness). Application of these principles provided guidance to adjust our design to the needs of our population and provided a level of conceptual clarity that linked our work with the behavior change literature at large. For example, although the term “notification” is common in mHealth, this feature is essentially an antecedent control strategy, which has been extensively used in the behavior change literature in areas such as autism [77-79], individuals with dementia and cognitive impairments [80-82], and in cases of traumatic brain injury and poor executive functioning [83,84].

Finally, our development effort took into account implementation considerations: (1) it used an Android operating system, which according to our panel was a common platform among people with SMI and tends to dominate the market among people with lower socioeconomic status (eg, individuals with disabilities) [85,86], and (2) it addressed the potential need for more intense personal support by including a technical coach feature within the app. In the future, this coaching role could be performed by addiction counselors or case managers in community mental health clinics and address not just technical issues but also support to use of ACT skills to deal with smoking cravings or adherence to USCPG.

### Comparison With Prior Work

The study reported in this paper is consistent with user-centered design research of mobile apps for depression [87], smoking cessation [30], and work with diverse populations [88]. Furthermore, Learn to Quit’s emphasis in gamification is consistent with an app developed for depression, SuperBetter [89].

To date, many apps have focused on the use of sensors and algorithms to track user context and provide personalized feedback [90-94]. Learn to Quit differs from this approach in the sense that it is based on a more traditional form of engagement, visual storytelling. Visual storytelling is a core form of engagement common across human cultures [95]. Storytelling and visual engagement are implicated in emotional arousal [96] and in attaching value and significance to sensory descriptions [97,98], thus contributing to learning and memory (eg, comprehension and retention of new content) [98,99]. Given the fact that motivational challenges are common among people with SMI (eg, negative symptoms of schizophrenia), we built an app that had visual storytelling at its core. Our main goal was to create an app with visuals and stories that were as engaging as possible, in combination with gamification, well-established behavior analytic principles, and the use of evidence-based smoking cessation content.

As stated in the introduction, we believe that the determination of the active therapeutic ingredients delivered by an app should be the result of a careful design process. However, user-centered design research could lead to stakeholder recommendations that are not consistent with evidence-based practices or theory-based principles of change. Adherence to evidence-based practices or theory-based principles of change might not always be emphasized in user-centered design research, yet it is a key activity of the design process inherent in the original user-centered design guidelines [31,32]. As shown in our Methods section, we emphasized this key activity by incorporating phases II and III and by making an effort to ensure that our final design integrated concerns brought up by each of these phases.

Finally, a relevant aspect of this app is that it could be a good example of the concept of universal design [100], for which systems tailored to specific groups of individuals with certain disabilities or challenges become inherently usable to larger groups of the population without those challenges. For example, even though the app addresses core usability barriers experienced in people with SMI, in doing so, it explains in simple terms very complex psychological concepts which might engage smokers of young age, low literacy groups, and the general public. Furthermore, the app’s systematic use of behavior analytic principles to increase comprehension and retention of ACT and USCPG is broadly applicable. Therefore, Learn to Quit’s design approach could be generalizable to related mental health conditions, health behaviors, or the general public.

### Limitations

This study had several limitations. First, the number of patients with SMI in the expert panel group (n=2) and the usability testing study (n=5) could have been small, leaving to question whether a larger sample of people with SMI could have led to more feedback and opportunities for innovation. There is debate among user-centered design researchers about the most cost-efficient number of subjects to identify usability errors [101,102], with the most traditional approach suggesting a sample size of 5 [101]. Recruiting individuals with SMI is particularly challenging; therefore, we believe our sample size was justified.

Second, our methods could have been more rigorous in several aspects. Specifically, results from our expert panel were not transcribed and analyzed using a complete set of qualitative methods, which could have led to the identification of additional themes. However, rather than a thorough and comprehensive analysis of provider input, the goal of this expert panel was to quickly gather initial insights and impressions that would orient our imminent design process. Additionally, usability testing did not include observational coding of user behavior. As reported in similar studies [30,87,88], this could have provided more concrete usability feedback and complement the results of the SUS and the thematic analysis of our transcripts. A third methodological limitation is that we did not conduct diagnostic interviews of our subjects. Despite this, all subjects received psychiatric treatment and were recruited from a community mental health clinic, which, according to regulations by the US Department of Health and Human Services [3], requires serving individuals with SMI status.

Third, the app’s tracking feature provided personalized feedback based on participants’ responses to self-reported ratings of mood and smoking behavior. However, this level of personalization did not take into consideration each individual’s baseline (eg, certain smoking reductions could be large or small depending on the individual’s baseline), limiting its impact for personalization. Likewise, the current app system does not take into account a variety of quitting scenarios (eg, individuals who quit before the end of the program) and how these scenarios interact with app content. Future versions could take into account these personal scenarios to strengthen Learn to Quit’s usefulness and level of personalization.

Finally, this paper focuses on the user-centered design research of a paper prototype leading to the development of the Learn to Quit app. Although this report does not provide data about the usability and user experience of the final Learn to Quit app, it allowed us to transparently report in more detail its user-centered design process. A separate report of Learn to Quit’s usability and user experience in its target population is under review elsewhere.

### Conclusions

This is the first paper to systematically describe the rationale, ideation, design, and user research of a smoking cessation app specifically designed for people with SMI, a population with alarmingly high rates of nicotine addiction and in high need of novel smoking cessation treatments. The feasibility and acceptability of this app will be subsequently tested in a randomized controlled feasibility trial (clinicaltrials.gov NCT03069482).

User-centered design is a critical process for the development of mobile interventions for individuals with SMI. However, because emotional and cognitive challenges are present in less severe forms of mental illness or can be present in other health conditions (eg, cancer patients), the results of this study might be generalizable to other areas of mHealth research. Therefore, while ideating and designing digital interventions, mHealth developers might consider capitalizing on the role of visual engagement, storytelling, and the systematic application of behavior analytic principles to deliver evidence-based content.

## Appendix

Multimedia Appendix 1 Primary and secondary personas that guided Phase IV of the user-centered design process.

Multimedia Appendix 2 Evidence-based smoking cessation content of Learn to Quit app.

Multimedia Appendix 3 Design principles.

## Abbreviations

ACT: Acceptance and Commitment Therapy
NCI: National Cancer Institute
SMI: serious mental illness
SUS: system usability scale
USCPG: US Clinical Practice Guidelines
