# Supplementary figures and images for: User-Centered Design of Learn to Quit, a Smoking Cessation Smartphone App for People With Serious Mental Illness
Source: JMIR Serious Games. 2018 Jan 16;6(1):e2. doi: 10.2196/games.8881 (PMC5790963; doi:10.2196/games.8881)

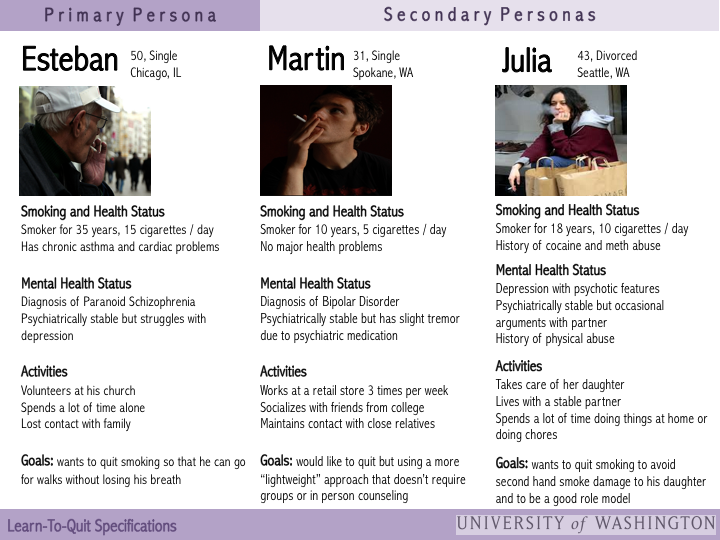

Supplement: Multimedia Appendix 1 [file games_v6i1e2_app1.png]
