# Supplementary material for: User-Centered Design of Learn to Quit, a Smoking Cessation Smartphone App for People With Serious Mental Illness
Source: JMIR Serious Games. 2018 Jan 16;6(1):e2. doi: 10.2196/games.8881 (PMC5790963; doi:10.2196/games.8881)
Supplement: Multimedia Appendix 2 [file games_v6i1e2_app2.pdf]

Appendix 1. Evidence-based Smoking Cessation Content of Learn to Quit app.

Level	Day	Track	Module (Component)	Content Description
Level I	1	Lesson	Finding Your North Star (Values Activation)	Orients the user towards the importance of identifying personal values for quitting and introduces the 1st core metaphor
	1	Skill	Your North Star For Quitting (Values Activation)	Implements a card sorting task to help the user identify 5 key values for quitting that will guide the user throughout the quitting journey
	2	Lesson	Smoking Poisons Your Body (USCPG)	Describes the health costs of smoking and the financial benefits of quitting
	2	Skill	Swamp of Urges (Awareness)	Guides the user to notice different bodily sensations while having an urge to smoke and introduces the 2nd core metaphor of the app
	3	Lesson	Urges Are Not Your Enemy (Openness)	Explains the difference between an urge to smoke and the behavior of smoking and casts the act of smoking as the problem
	3	Skill	Let's Pretend Urges (Openness)	Prompts the user to visualize urges as having physical properties (e.g., color, shape)
	4	Lesson	The Key To Quitting (Openness)	Encourages the user to change their relationship with urges and consider them as a "friend"
	4	Skill	Be Mindful Of Your Feelings (Awareness)	Introduces the role that emotions play in smoking cessation and guides the user to notice their emotional responses while having an urge to smoke
	5	Lesson	Open Your Senses (Awareness)	Expands the concept of awareness of urges to include contact with experiences from our 5 senses
	5	Skill	Use Your Five Senses (Awareness)	Guided exercise that prompts her to experience their 5 senses
Level II	6	Lesson	Nicotine Patch and Lozenges (USCPG)	Discusses the importance of nicotine replacement therapy to help quit smoking
	6	Skill	A Little Patch (USCPG)	Helps the user minimize a common adverse event of nicotine patches (i.e., skin irritation) by helping identify different areas of the body where patches could be applied
	7	Lesson	Be Willing To Have Urges (Openness)	Introduces the concept of willingness to have urges as key to quitting smoking
	7	Skill	Be Mindful Of Your Thoughts (Awareness)	Offers an exercise to increase awareness of smoking triggers with a focus on cognitive content
	8	Lesson	Smoking Is Not Who You Are (Openness)	Introduces a distinction between the user's nuanced identify (i.e., "a person") and their simplified identify (e.g., "a smoker")
	8	Skill	Mind and Feet (Openness)	Offers an exercise that empowers the user to stay smoke free during episodes of intense cognitive triggers to smoke
	9	Lesson	Commit To A Quit Date (USCPG)	Describes the importance of setting up a quite date and encourages the user to set up their personal quit date
	9	Skill	Your Valued Actions (Values [illegible]	Helps identify 5 specific activities that are consistent with one of the values stated by the [illegible]
