# Supplementary material for: User-Centered Design of Learn to Quit, a Smoking Cessation Smartphone App for People With Serious Mental Illness
Source: JMIR Serious Games. 2018 Jan 16;6(1):e2. doi: 10.2196/games.8881 (PMC5790963; doi:10.2196/games.8881)
Supplement: Multimedia Appendix 3 [file games_v6i1e2_app3.pdf]

Table 4. Design Principles

Principle	Description	Implementation
Learning Principles		
Differential reinforcement of successive approximations[70]2]	A skill is gradually shaped across successive trials by rewarding smaller segments of that behavior	Smoking cessation skills are broken down into smaller units, gradually presented and reinforced using game rewards
Multiple Exemplar Training [61]	Variations of a given concept or skill are deliberately used to increase learning generalizability	Multiple examples of smoking cessation concepts designed to increase the generalization of such skills to a variety of settings
Positive Reinforcement **[70]2]**	Consequences are presented to increase the frequency of a given behavior	Completion of app modules followed by gaming rewards to increase their frequency
Fixed ratio of reinforcement [70]2]	A reward is presented after a specified number of responses to produce a steady rate of responding	Completion of app modules followed by a fixed scheme of gaming rewards (e.g., 5 points after each module)
Antecedent Control **[72]4]**	Certain stimuli are presented to prompt an already stablished response	Use of app notifications and visual animations to trigger app behavior
Negative Reinforcement [70]2]	Consequences are removed to increase the frequency of a given behavior	Removal of app rewards if user does not complete certain modules or displays certain app behaviors
Design Principles to address SMI		
Minimizing Cognitive Load[30,38,39,41]6]	Minimizing the amount of semantic information to be processed by the user	Brief app content with minimal words and 6th grade level sentences; predictable format and wireframes structure
Maximizing Visual Acuity[14]2]	Increase in discrimination of letters, numbers and symbols at a certain distance	Large typeface, numbers and icons to maximize visual processing and minimize touchscreen errors
Flat Design[73]6]	Minimalist use of stylistic elements, such as typography, and colors	Simple and consistent visual palette across wireframes and lack of dynamic screen elements (e.g., hiding menus)
Minimal Layer Structure[38]3]	Arrangement of app wireframes so that access to available content requires a minimal number of steps	Only 2 steps are required to access any available app content or features
Storytelling	The use of stories and interactive characters to convey concepts or encourage the use of skills	App content is embedded within an overarching narrative that includes characters and storytelling elements
